# The construction and practice path of safety education mechanism in colleges and universities integrating the psychological characteristics of students in the new era

**DOI:** 10.3389/fpsyg.2022.1056021

**Published:** 2023-01-06

**Authors:** Honglin Li

**Affiliations:** Beijing Institute of Fashion Technology, Beijing, China

**Keywords:** college safety education, recommendation algorithm, online learning and testing system, psychological characteristics of students in the new era, practice approach

## Abstract

**Background:**

With the rapid development of higher education in China, the scale of colleges and universities is expanding, and the phenomenon of campus socialization is becoming more and more obvious. In particular, the campus and its surrounding environment are becoming more and more complex, which brings many hidden dangers in university life.

**Objective:**

In order to improve the effectiveness of safety education in colleges and universities and maintain the long-term effectiveness of college students’ safety awareness, the paper proposes the construction and practice path of college safety education mechanism that integrates the psychological characteristics of students in the new era.

**Methods:**

Security issues facing universities at home, this track identifies the relationship between campus security incidents and security education and advocacy. Eight solutions to prevent and reduce incidents in schools. The paper proposes to give importance to the study of the security of college students, to create an awareness of security questions in the bank based on the recommendation algorithm, and to create to have online learning and testing for safety awareness.

**Results:**

The passing rate of 10 majors such as humanities, composition and theory of composition technology was 100%, accounting for 12% of the 83 enrolled majors, and the passing rate of 54 majors such as clinical medicine was over 90%.

**Conclusion:**

The safety online learning and testing system of college students’ safety education is lively in form and highly accepted by students. The development of college students’ safety education starts from the time of receiving the university admission notice, making full use of the “golden time,” so as to effectively prevent and reduce the occurrence of campus safety accidents.

## 1. Introduction

Security is the premise of social development, the guarantee for the survival and development of individual human beings, and the focus of human attention (Goode et al., 2021). Today’s society is a rapidly developing and open society, the living space of college students is greatly expanded, and the field of communication is also constantly expanding. With the rapid development of higher education in China, the scale of colleges and universities is expanding, and the phenomenon of campus socialization is becoming more and more obvious. In particular, the campus and its surrounding environment are becoming more and more complex, which brings many hidden dangers in university life. In recent years, security incidents in colleges and universities have occurred from time to time, and the security of college students has begun to attract great attention from all walks of life. As we all know, college life is a critical period in the life of college students, and the safety factor will directly affect the quality of their study and life in college, and will even be the key factor for college students to become adults, talents, and success. Therefore, strengthen the safety education and management of colleges and universities, improve the safety awareness and skills of college students, improving the safety awareness of college students is a simple course to ensure the physical, and mental safety of college students. It is not only an important part of a good education for college students, but also an important element of ideological and political education in colleges and universities.

**Figure 1:**
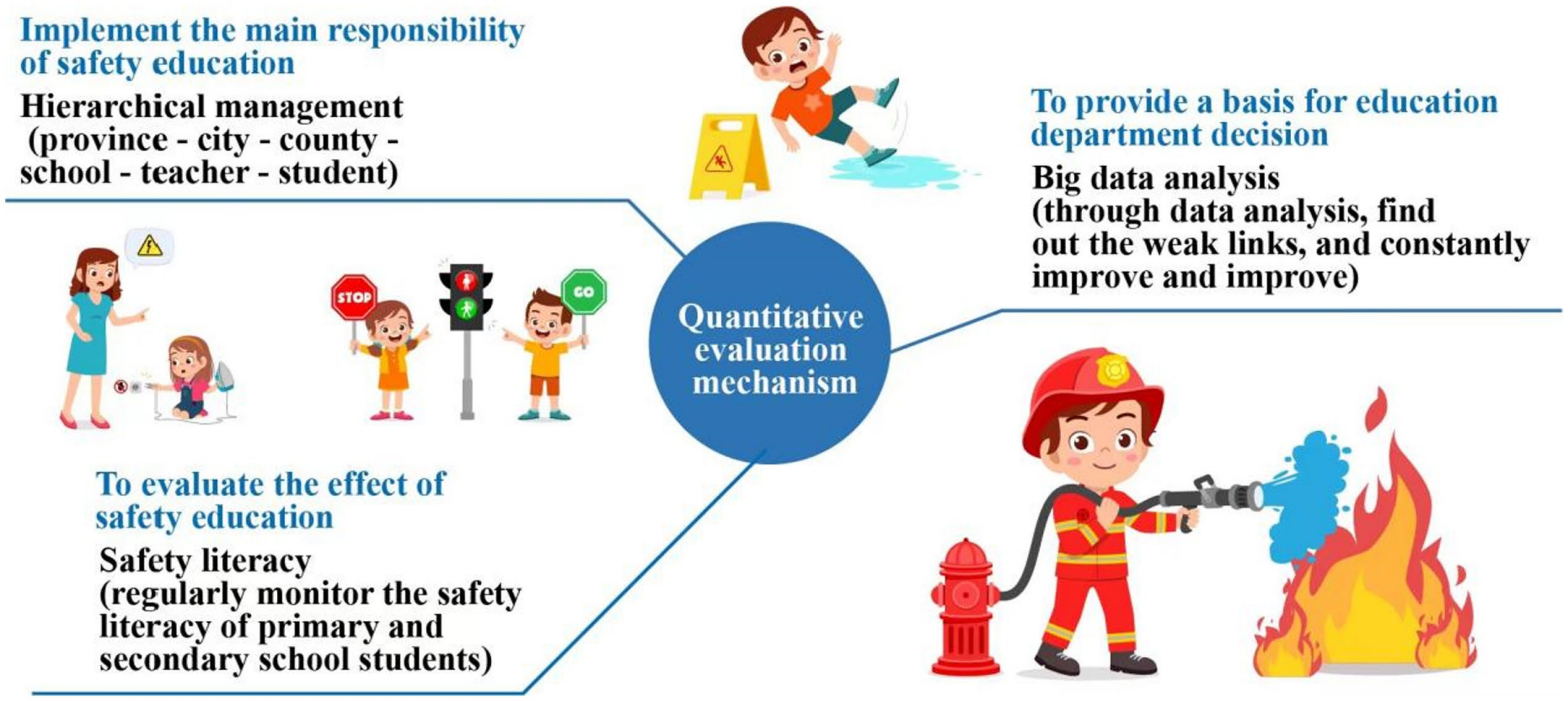
Safety education management and supervision mechanism.

At present, the standard of safety education for college students is single, and the new safety education does not include the survival and rescue of life knowledge, emergency education and network security (Wang et al., 2021). Safety education is not a profession and most colleges and universities focus only on providing safety knowledge, lack of safety training, best practice, poor quality and lack of safety education when first year students are enrolled (Demello et al., 2020). In many colleges and universities, security is not enough, and security and support systems and processes are simple, broad, and lacking in depth. Safety education is based on modern and modern tools such as publications and manuals. But ignores the knowledge and experience of universities. Nowadays, the major students in high schools and universities are the post-1995 and post-2000 generation (Wu et al., 2021). It is impossible to attract these students, most of the students close their eyes, and safety promotion does not use all the new media such as APP, WeChat, Weibo, and other, and it is not safe in school culture. Figure 1 shows the safety management and control system (Marques et al., 2021).

College students should not only arm their minds with scientific knowledge, but also observe society with a keen eye, establish a scientific world outlook, outlook on life, and values, and handle the relationship between knowledge, intelligence, quality, and spirit (Faleide et al., 2021). Therefore, quality, especially safety quality, is the foundation and guarantee of all this. “People-oriented” is the main concept of safety culture education in colleges and universities, and safety education is the starting point and foundation. In order to realize the unity of social and personal values of morality, moral education to people cannot be done without safety education. Creating a safe and stable environment is conducive to the development of individuals. Therefore, the healthy and comprehensive development of college students is inseparable from safety education and management.

With the rapid development of China’s higher education, colleges and universities have transformed from the original closed teaching to open teaching, and Chinese colleges and universities have undergone profound changes, these changes not only bring vigor and vitality to the development of colleges and universities, but also bring new problems and challenges to school safety management (Feng et al., 2020). At the same time, with the rapid development of the economy, the application of various media, and networks has broadened the channels for college students to receive information, some unhealthy and negative information in the society is introduced into the campus through various channels, which has a negative impact on college students. Especially in today’s rapid development of information technology, computers and mobile phones have entered 1000’s of households. However, as the existence of high technology in this century, mobile phones and the Internet have their extremely advanced aspects and also have many negative effects, such as pornographic images, gambling scams, unhealthy chats, online games, etc., some college students use mobile phones or the Internet to commit crimes. Therefore, we must strengthen the safety education of college students, maintain the safety and stability of the campus, create a good environment for teaching and educating people, and ensure the safety and smooth progress of the study and life of college students.

At present, China’s higher education is gradually being popularized, and the scale of college enrollment has been different from the past. University campuses have gradually changed from a closed “ivory tower” to an all-round, multi-functional and open “small society.” At the same time, most colleges and universities are now open schools where outsiders can enter and leave the campus at will, and criminals will seize the opportunity to enter the campus for illegal criminal activities such as fraud and theft, consequently adding much uncertainty to the risk of school security. Facing the current complex situation, freshmen are not aware of the danger, weak security awareness, and even give criminals an opportunity. Therefore, there are theft caused by poor personal custody, goods littering, dormitory nobody and not locking doors and windows, and lack of appropriate experience and methods.

Colleges and universities are the cradles of cultivating talents, and are places where intellectuals and talents are concentrated, in a sense, the stability of colleges and universities is directly related to the stability of people’s hearts and the smooth progress of economic construction (Brungart et al., 2020). Security has evolved into a comprehensive concept, and its content has expanded from military and political to economic, technological, environmental, cultural and other fields (Wang et al., 2020). Secondly, with China’s economic development, social stability, people living and working in peace and contentment, its international status is increasing day by day, the peaceful environment makes college students relax their vigilance against the sabotage activities of hostile forces at home and abroad, and weaken their security awareness.

## 2. Materials and methods

### 2.1. Construction of online learning and testing system for student safety education

The creation of online security training and testing must first create awareness of bank security, which is an important task. The university’s security education online training and test bank questions are divided into two types: Knowledge bank questions and bank questions; These types usually include two types: multiple choice, and true and false questions (Ealy and Stauffer, 2020).

General knowledge usually includes knowledge of public safety, knowledge of fire prevention, fraud, road safety, food safety, laboratory safety, national security, etc., and account for about 60% of all banking inquiries; Meanwhile, the expansion of these knowledge points is as important as the type of crime at a particular school (Chiang et al., 2020). In the database, since 2010, most universities and schools, especially those related to Qingdao University, the process of writing questions to teach students safety skills is multi-purpose and functioning and has educational benefits for students.

In order to make the questions about the knowledge multi-purpose, the recommended content as the system was created, comparing different questions for different groups of experts and different characteristics of users.

Determine the personal vector of the student and the type of question, according to X and Y, the product of the personal vector of the student is work, education, high level, knowledge level, etc. The question type feature vector contains knowledge base category expertise, level of security knowledge, and coverage of knowledge content.

Assuming that the i-type questions that students have done on the mth day are $l_{i}$, $a_{\text{mj}}$ and $b_{\text{mj}}$ respectively represent the score and total score of the j-th question, then the user’s total score $M_{i}$ and total score $N_{i}$ of the i-type questions on this day are respectively:


(1) $$M_{i}=\sum_{j=1}^{l_{i}}\theta \times a_{mj}$$

(2) $$N_{i}=\sum_{j=1}^{l_{i}}\theta \times b_{mj}$$

Among them, θ is the difficulty level of the question, which has three values, representing the weights of difficult, medium and easy, respectively.

Calculate the student’s score for the question type based on the students’ answers:


(3) $$X_{i}=\frac{\sum_{m=1}^{t}\left(\frac{1}{\log8m}\times M_{i}\right)}{\sum_{m=1}^{t}\left(\frac{1}{\log8m}\times N_{i}\right)}$$

The student eigenvector is calculated by formula (3), and the initial vector is 0.

The quantitative value of each of the query-type feature vector is to extract the feature vector based on the previously established knowledge base security features, such as the knowledge in the professional level, level security awareness, and content awareness.

Calculate the similarity between students and questions by calculating the two eigenvectors of students and question types.


(4) $$d\left(x,y\right)=\sqrt{\sum {\left(x_{i}-y_{i}\right)}^{2}}$$

Based on the similarity matrix, relevant questions are selected and applied to the user (Kulinski et al., 2020).

Online security surveys and tests are written using hypertext preprocessor and dynamic web pages are very efficient. The data system is integrated into MySQL, which stores data in several tables, which speeds up the processing and queries and improves convenience. The SQL language used by MySQL is the most widely used language for accessing data, and its small size and speed ensure that many users can access it at the same time.

An online safety report and test to support the integration of automatic scorers and instructor scorers. There are four learning and testing methods available: retraining, experimental testing, operational testing, and non-paper work. Contestants’ details can be entered in one click and match scores can be exported in one click. If there is no time and space restrictions, all online activities save a lot of resources, such as personnel and materials. The system is hosted on the university’s server and provides access to other networks so that candidates can access the system on time for online courses and exams across the country.

### 2.2. Standardize management and innovate the institutionalization of safety education for college students

The establishment of a sound school safety education and management organization is the basic guarantee for the normal operation of safety education for college students. The safety education work in colleges and universities must be strictly managed, implement the responsibility, and set up a special organization to lead, organize and coordinate the school safety education work. Safety education should be in line with the principle of “who is educated; who is educated, who is in charge, who is responsible,” should take prevention first, in line with the principle of people-oriented, teacher-student protection, education first, clear responsibility, seeking truth from facts, and do a good job in safety education. Establish safety education teaching and research institutions in colleges and universities, formulate feasible teaching plan, buy college students’ safety education materials, hire law, public security, fire control, relevant leadership, security department comrades combined with specific cases of the legal system, fire safety, escape from education, it has a positive role in preventing the occurrence of safety accidents. In addition, we should give full play to the advantages of their own school, the safety education into the teaching plan. Make it standardized, institutionalized, safety education and other public disciplines with the arrangement, layout, with assessment, with evaluation.

### 2.3. Due to the timing of teaching, improve the construction of safety education teachers in colleges and universities

With the expansion of college enrollment scale and the increase of college students, the task of college safety education and management is becoming more and more difficult. The safety problems of college students occur from time to time, and the safety situation of college students is not optimistic. At present, the safety education work of college students is basically undertaken by the security department staff and political counselors, the security department workload, complicated affairs; in addition to the daily complex students thought, study and life work, counselors also constantly strengthen their academic study and consider the future problems, so that the student safety education work is weak, even if the safety education content is mostly stay in oral preaching, lack of education practice. Therefore, colleges and universities in strengthening the construction of safety education work team, should mobilize the strength of the functional departments of the school, through the school propaganda department, academic affairs office, student affairs department, security department and other departments of the coordination, mobilize the head teacher, class teacher to carry out targeted safety education for college students. Strengthen the construction of the full-time team of safety education for college students, improve the political quality of the safety education team, guide the students to strengthen the vigilance of the sabotage activities of the hostile forces at home and abroad, and correctly treat the domestic and international hot and difficult issues. At the same time, strengthen the safety education team business training, enrich safety knowledge, skilled in fraud prevention, fire prevention and other safety skills, adhere to the “prevention first, fighting and prevention combined.” Strengthen safety education and publicity, teach students in accordance with their aptitude, and improve the enthusiasm of students to learn safety knowledge and skills. According to the survey, many colleges and universities have pushed the task of safety education and management to the security departments, student engineering departments and student counselors, and have not set up full-time safety education teachers. To do a good job in safety education and management, it is far from enough to rely only on these teachers alone. Therefore, the construction of teachers to strengthen the safety education and management is necessary to ensure the quality of safety education of college students.

### 2.4. Experimental subject

**Figure 2:**
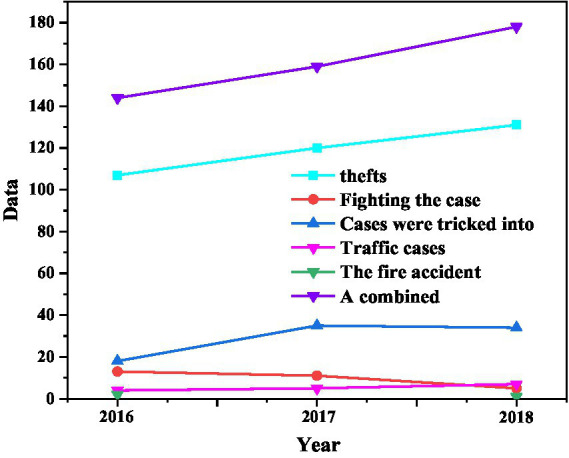
Summary of the number of security incidents in a university from 2016 to 2018.

Using schools as examples, see below for student safety violations for 2016–2018. Based on Tables 1–4, theft is the most common, accounting for 70% of security crimes every year (Bai et al., 2021); The location of the theft, most likely to be classrooms and dormitories, is based on student membership and will often be divided into classrooms and dormitories while students were in residence. There are few hotel staff in the rooms, which allows thieves to take advantage. This is because they did not take the information away when studying for free in the classroom. Since its release, they usually occur in June and September every year, June is the exam season and graduation season, students like to focus on their exams and prepare for them. As shown in Figure 2, student theft has increased from 2016 to 2018, indicating that college students’ security awareness has decreased to this level after changes in community. The school did not implement the safety training on time, and the safety training is not mandatory.

**Table 1:** Statistics on the occurrence of security incidents in a university in 2016.

Month		1	2	3	4	5	6	7	8	9	10	11	12	Total
Incident of theft	Dormitory	2	/	2	2	3	6	1	2	3	2	1	1	25
	Classroom	8	/	2	3	5	6	5	/	8	2	/	2	41
	Dining room	1	/	/	/	/	6	1	/	/	3	/	/	5
	Library	/	1	1	/	2	/	1	/	/	2	/	/	6
	Stadium	/	/	/	1	1	2	1	/	3	1	/	/	9
	Waterhouse	/	/	/	/	/	1	/	/	/	1	/	/	2
	Bathhouse	/	/	/	1	/	1	/	/	/	1	/	/	3
	Other	/	1	/	/	/	1	3	1	5	2	1	2	16
Subtotal		11	2	5	7	11	17	11	3	20	13	2	5	107
Brawl case		1	/	/	/	1	5	11	1	2	/	1	/	13
Deceived case		/	/	1	/	3	/	2	/	6	4	2	2	18
Traffic case		/	/	1	/	1	/	/	/	2	/	/	/	4
Fire accident		/	/	/	1	/	/	1	/	/	/	/	/	2
Total		12	2	7	8	16	22	14	4	30	17	5	7	144

**Table 2:** Statistics on the occurrence of security incidents in a university in 2017.

Month		1	2	3	4	5	6	7	8	9	10	11	12	Total
Incident of theft	Dormitory	/	/	1	1	2	7	1	/	2	7	3	7	31
	Classroom	2	1	3	5	4	7	3	/	11	5	3	3	47
	Dining room	/	/	1	/	2	1	/	1	3	/	1	/	9
	Library	/	/	1	1	2	2	/	/	1	/	/	/	7
	Stadium	/	/	/	1	/	1	/	/	2	1	1	/	6
	Waterhouse	/	/	/	/	/	1	/	/	2	1	/	/	4
	Bathhouse	1	/	/	/	/	/	/	/	/	/	1	/	2
	Other	1	/	1	/	1	2	/	1	3	2	1	2	14
Subtotal		4	1	7	8	11	21	4	2	24	16	10	12	120
Brawl case		/	1	/	2	1	3	1	1	1	/	/	1	11
Deceived case		/	2	2	3	1	2	/	1	6	2	3	1	23
Traffic case		1	/	/	/	/	1	1	/	1	1	/	/	5
Fire accident		/	/	/	/	/	/	/	/	/	/	/	/	0
Total		5	4	9	13	13	27	6	4	32	19	13	14	159

**Table 3:** Statistics on the occurrence of security incidents in a university in 2018.

Month		1	2	3	4	5	6	7	8	9	10	11	12	Total
Incident of theft	Dormitory	3	/	1	3	4	6	4	1	5	4	2	2	35
	classroom	4	/	2	5	7	6	4	3	9	6	2	3	51
	Dining room	/	/	1	1	1	1	/	/	/	1	2	2	9
	Library	/	/	3	/	1	/	/	/	2	/	1	1	8
	Stadium	/	/	/	/	/	/	2	/	2	/	/	/	4
	Waterhouse	/	/	/	/	/	/	1	/	/	1	1	1	4
	Bathhouse	/	/	/	1	/	/	/	/	/	2	/	/	3
	Other	/	1	/	1	1	3	2	/	5	2	1	1	17
Subtotal		7	1	7	11	14	16	13	4	23	16	9	10	131
Brawl case		/	/	/	/	1	2	/	/	/	1	/	1	5
Deceived case		1	/	3	2	1	2	1	2	4	8	5	5	34
Traffic case		/	/	1	/	/	1	/	/	1	2	1	1	7
Fire accident		/	/	/	/	/	/	/	/	/	1	/	/	1
Total		8	1	11	13	16	21	14	6	28	28	15	17	178

**Table 4:** Statistical summary of security incidents in a university from 2016 to 2018.

Month		2016	2017	2018
Incident of theft	Dormitory	25	31	35
	Classroom	41	47	51
	Dining room	5	9	9
		6	7	8
	Library			
	Stadium	9	6	4
	Waterhouse	2	4	4
	Bathhouse	3	2	3
	Other	16	14	17
Subtotal		107	120	131
Brawl case		13	11	5
Deceived case		18	35	34
Traffic case		4	5	7
Fire accident		2	/	1
Total		144	159	178

“/” stand for not determined/not available.

From 2016 to 2018, there has been a clear upward trend in student cheating incidents and on-campus traffic accidents, which is related to the rapid advancement of network technology in recent years to provide space for online fraud, and the increase in social vehicles has greatly increased the number of campus accidents, among the school security incidents against students, the theft has the highest probability of occurrence, accounting for 70 percent of the annual security incidents (Jna et al., 2020). There are no students who are taught well.

In order to analyze the correlation between the safety education level E and the accident rate A. The Pearson Correlation Coefficient (method, quantitative analysis of the correlation between variables) was used.

## 3. Results

In order to analyze the correlation between the safety education level E and the accident rate A (Olyunin et al., 2021). The Pearson Correlation Coefficient (method, quantitative analysis of the correlation between variables) was used (Wang X., 2021). Pearson correlation analysis PCC formula:


(5) $$\rho_{E,A}=\frac{\mathrm{cov}\left(E,A\right)}{\sigma_{E}*\sigma_{A}}=\frac{\sum EA-\frac{\sum E\sum A}{N}}{\sqrt{\left(\sum E^{2}-\frac{{\left(\sum E\right)}^{2}}{N}\right)\left(\sum A^{2}-\frac{{\left(\sum A\right)}^{2}}{N}\right)}}$$

Among them, the variable N is the number of objects under investigation, with the college as the unit; The safety education level E is defined as the student’s safety education achievement, and the accident rate is defined as the frequency of student safety incidents (Wang G., 2021).

According to the distribution of campus safety accident data in various colleges in the above 3 years, the data of 13 colleges were extracted, and the average scores of safety education in each college were compared, the correlation coefficient between safety education E and safety accident rate A from 2016 to 2018 is calculated from formula (5), as shown in Table 5.

**Table 5:** The correlation between safety accidents and safety education in various colleges of a university from 2016 to 2018.

	Years	2016		2017		2018	
College		E	A	E	A	E	A
School of chemistry and chemical engineering		92	6	95	8	90	9
Faculty of physical sciences		90	11	88	15	92	12
Mechanical and electrical engineering		95	5	92	8	91	7
School of electrical engineering		90	8	93	11	91	10
Textile and clothing college		88	13	90	12	85	16
Electronic information school		83	16	85	13	83	15
School of history		95	7	88	10	90	11
School of foreign languages		82	15	80	18	88	14
Business school		89	13	84	12	82	15
Art college		92	9	91	8	90	10
School of economics		90	7	90	10	91	9
basic medical school		92	7	91	8	88	10
College of pharmacy		93	8	94	6	94	5
ρE·A		−0.89		−0.83		−0.84	

From the data and analysis in Tables 1–5, there are certain regularities in the campus security incidents that occur to students, regardless of the location, time period, nature and type of the incident. Taking the 2019 freshmen of a university as an example, the pass rate of the student test is 91.25%, and the pass rate of 10 majors such as humanities, composition and composition technology theory is 100%, it accounts for 12% of the 83 majors enrolled, and the pass rate of 54 majors including clinical medicine exceeds 90%. Among the school security incidents against students, the theft has the highest probability of occurrence, accounting for 70 percent of the annual security incidents. If there are regular security incidents, it is possible to reduce the occurrence of security incidents by taking measures such as strengthening student safety education, strengthening campus and building door management, and strengthening campus patrols, among them, safety education is the most effective and easy to popularize feasible method.

**Figure 3:**
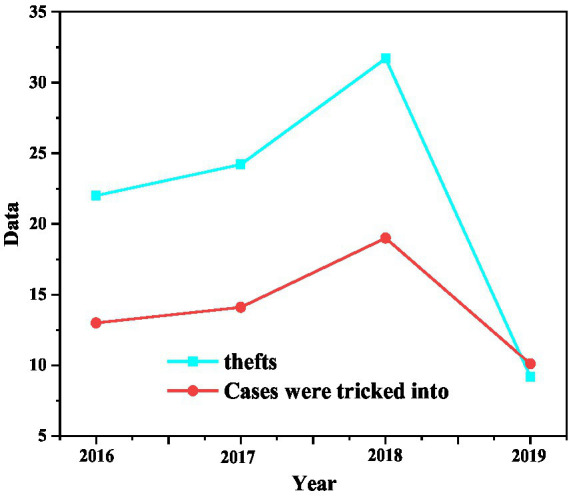
Comparison of the number of security incidents during the freshman year of 2016 to 2019.

Through the 2016, 2017, 2018 and 2019 freshmen within 1 year after entering the school, statistical comparison of student theft and deception reports (Figure 3), it can be seen that these two numbers of 2019 students have shown a significant downward trend in the 2018, this proves that the safety education of college students has some impact, the dissemination of new safety knowledge and the development of safety skills have some influence (Chen et al., 2020).

## 4. Discussion

Taking the freshmen of 2017 in a university as an example, 91.25% of the students passed the test, and the pass rate of 10 majors such as humanities, composition and composition technology theory, accounting for 12% of the 83 enrollment majors, and the pass rate of 54 majors including clinical medicine was more than 90%. Through the 2014, 2015, 2016 and 2017, the four freshmen a year after the school, student theft and fraud report statistics, know, 2017 students a year the two figures obviously lower trend, proved that based on the “Internet 10” college students safety education front has achieved obvious effect, the popularization of new security knowledge and the improvement of security skills have obvious results.

The contradiction between teachers and students is the basic contradiction in the process of school education and teaching, which is normal and inevitable under certain conditions. However, in the face of contradictions, if the teachers and students do not take reasonable, legal and peaceful means of communication, but adopt extreme words, expressions and behaviors, the contradictions between teachers and students will intensify, the relationship between teachers and students will deteriorate, and the contradictions will evolve into conflicts. In the face of the conflict between students and teachers, teachers feel helpless. This is because more and more people who pay attention to education and research education but do not engage in front-line education blame teachers for all the problems arising in the process of education and all the contradictions between teachers and students, and even think that “There are no bad students, only bad teachers.” The teachers just smiled helplessly. In essence, the existence of the contradiction between teachers and students is objective and inevitable, but the contradiction between teachers and students is not absolute, but relative. Every responsible teacher loves his own students. “Jade, not,” “strict” this is the teacher in the process of education students adhering to the basic ideas, at the same time, every student wants to become useful person to the country and society, from the starting point, teachers and students are consistent, so, why will conflict between teachers and students? In my opinion, the conflict in the teacher-student relationship is mainly due to the misunderstanding in the process of communication between teachers and students. Teachers fail to understand the psychological state of the students well, and fail to make the students feel their love for them in an appropriate way. Therefore, suggest that the teachers, who occupy the dominant position in the teacher-student relationship, should better grasp the psychological state of the students, and actively promote the harmonious development of the teacher-student relationship. The starting point of safety education is the “people-oriented” education concept, the ultimate foothold is to maintain the national, social, campus law and moral order, cultivate students to catastrophic accidents, emergencies, daily dangerous activities of emergency treatment and strain ability, safeguard staff and students’ personal, property and ideological security, the escort for the teaching task smoothly.

In the age of risk, safety education in universities is no longer limited to the physical safety and security of students, but also needs to take into account psychological monitoring and property security. Particularly in the Internet Age, where Internet fraud has proliferated, students are constantly receiving phone calls and text messages even when they are on campus, and the scams are escalating. The theme and focus of cyber security education in higher education should be in line with the pace of the times, and online evaluation is an effective measure.

## 5. Conclusion

The paper proposed ways to create and implement safety education methods in colleges and universities that integrate the psychological characteristics of students new age, and scrapped safety curriculum as a prerequisite for safety education for college students in some countries, and it is still difficult to navigate the rigorous course of study required at various colleges and universities, it increases the novelty and effectiveness of safety education content, and also provides certain interactive functions, students can ask questions with the help of the system platform, the school safety management department can also release relevant safety knowledge in the form of notices, announcements or WeChat push according to the current focus of safety management, which improves the timeliness of safety education. Based on the analysis above, online training and testing for safety education of college students is more powerful and the enrollment of students is high, making full use of “shift hours” in order to prevent and reduce school safety incidents. Taking college freshmen in 2019 as an example, the pass rate of students was 91.25%, the technical theory pass rate of 10 disciplines including humanities and composition was 100%, accounting for 12% of 83 majors, and the pass rate of 54 majors such as clinical medicine exceeded 90%. Among school security incidents for students, theft has the highest probability, accounting for 70% of annual security incidents. Effectively improve the safety education in colleges and universities, and maintain the long-term effectiveness of college students ‘safety awareness. In the future, we can continue to study the safety education knowledge evaluation module by building the evaluation questionnaire with other colleges and universities, and conduct dynamic evaluation on students’ safety awareness.

## Data availability statement

The original contributions presented in the study are included in the article/supplementary material, further inquiries can be directed to the corresponding author.

## Author contributions

HL: conceptualization and formal analysis, investigation, writing original draft, and writing–review and editing.

## Funding

This work was supported by Beijing Institute of Fashion Technology General Subject (2020), the name of the project is “Triple Improvement” Safety Education in Colleges and Universities, and the grand number is DJ2020-01.

## Conflict of interest

The author declares that the research was conducted in the absence of any commercial or financial relationships that could be construed as a potential conflict of interest.

## Publisher’s note

All claims expressed in this article are solely those of the authors and do not necessarily represent those of their affiliated organizations, or those of the publisher, the editors and the reviewers. Any product that may be evaluated in this article, or claim that may be made by its manufacturer, is not guaranteed or endorsed by the publisher.
